# Ancient lineage, young troglobites: recent colonization of caves by *Nesticella* spiders

**DOI:** 10.1186/1471-2148-13-183

**Published:** 2013-09-04

**Authors:** Yuanyuan Zhang, Shuqiang Li

**Affiliations:** 1 Key Laboratory of Zoological Systematics and Evolution, Institute of Zoology, Chinese Academy of Sciences, Beijing 100101, China; 2 University of Chinese Academy of Sciences, Beijing 100049, China

**Keywords:** Biogeography, Araneae, Troglobites, Yunnan–Guizhou Plateau, BPP, *BEAST

## Abstract

**Background:**

The evolution and origin of cave organisms is a recurring issue in evolutionary studies, but analyses are often hindered by the inaccessibility of caves, morphological convergence, and complex colonization processes. Here we investigated the evolutionary history of *Nesticella* cave spiders, which are mainly distributed in the Yunnan–Guizhou Plateau, China. With comprehensive sampling and phylogenetic and coalescent-based analyses, we investigated the tempo and mode of diversification and the origins of these troglobites. We also aimed to determine which factors have influenced the diversification of this little-known group.

**Results:**

Coalescent-based species delimitation validated the 18 species recognized by morphological inspection and also suggested the existence of cryptic lineages. Divergence time estimates suggested that *Nesticella* cave spiders in the Yunnan–Guizhou Plateau constituted a monophyletic troglobite clade that originated in the middle Miocene (11.1–18.6 Ma). Although the Yunnan–Guizhou Plateau clade was composed exclusively of troglobite species, suggesting an ancient common subterranean ancestor, we favor multiple, independent cave colonizations during the Pleistocene over a single ancient cave colonization event to explain the origin of these cave faunas. The diversification of plateau *Nesticella* has been greatly influenced by the sequential uplift of the plateau and likely reflects multiple cave colonizations over time by epigean ancestors during Pleistocene glacial advances.

**Conclusions:**

We concluded that plateau cave *Nesticella* represent an ancient group of spiders, but with young troglobite lineages that invaded caves only recently. The absence of extant epigean relatives and nearly complete isolation among caves supported their relict status. Our work highlights the importance of comprehensive sampling for studies of subterranean diversity and the evolution of cave organisms. The existence of potentially cryptic species and the relict status of *Nesticella* highlight the need to conserve these cave spiders.

## Background

Cave organisms have long intrigued biologists, who have studied their general ecology, adaptations, and taxonomy, with focuses on the origins of troglobites (obligate cave dwellers) and their adaptation to specialized cave life [1-4]. The subterranean environment is characterized by permanent darkness, a lack of diurnal and annual rhythms, and a shortage of energy sources [5-7]. Animals, especially terrestrial invertebrates, adapted to cave environments are often reported to be highly geographically isolated, because of their limited dispersal ability resulting from limited physiological tolerances [8-10]. Their spatial isolation, simple community structures, and habitat specialization make them excellent model systems for studying evolutionary, biogeographic, and ecological issues.

The competing climate-relict and adaptive-shift hypotheses have been proposed to explain the origins of cave organisms [3]. According to the climate-relict model, pre-adapted epigean ancestors took refuge in caves when the surface climate was altered by glaciation or aridification and gradually adapted to the cave environment. Climatic oscillations caused local extinctions of surface populations, leaving each relict population to evolve allopatrically in a separate cave system [11,12]. The adaptive-shift model supposes that pre-adapted epigean species actively colonized caves to exploit novel resources and diverged under a gene flow scenario [13,14]. Divergent selection among epigean and subterranean habitats gradually overcame the homogenizing process of gene flow and eventually led to parapatric speciation [15].

A group of cave organisms could be the product of a single colonization of the subterranean habitat by an epigean ancestor, and the resulting phylogenetic pattern would be a sister relationship between the epigean species and the cave lineage. Alternatively, if each cave population had an epigean sister population in the tree, which would suggest that epigean ancestors colonized caves multiple times independently. Recent molecular phylogenetic studies of cave species usually recover a monophyletic lineage throughout a (usually large) geographical area composed exclusively or primarily of subterranean taxa [16,17]. This phylogenetic pattern indicates that ancestors of the subterranean lineage evolved a troglobitic lifestyle and that extensive dispersal happened after the cave colonization [16,17]. However, this conclusion contradicts the generally recognized limited dispersal ability of cave organisms, especially terrestrial cave invertebrates.

The South China karst represents one of the world’s most spectacular examples of temperate to subtropical karst landscapes. It was listed as an UNESCO World Heritage Site in 2007. Numerous cave-dwelling species inhabit the many highly isolated caves [18], including spiders of the genus *Nesticella* (Nesticidae), which currently includes 38 described species [19]. *Nesticella* is a cosmopolitan genus of small, sedentary, web-building spiders frequently found in leaf litter, debris, houses, and caves [20]. Over the past 7 years, we have conducted an extensive survey of these species in the Yunnan–Guizhou Plateau. More than 1,400 caves and their surrounding surface regions were visited, and we ultimately sampled 100 *Nesticella* populations. Most of these populations represented endemic species, and multiple populations of the same species were often sampled. With one exception, these *Nesticella* were exclusively found in caves; the cosmopolitan *Nesticella mogera*, however, occurs in both caves and surface habitats. The phylogenetic relationships between this widespread surface species and its troglobite relatives have not been investigated previously. Furthermore, two well-defined geological events allowed us to calibrate the phylogeny and provide a temporal framework for the evolutionary history of *Nesticella*.

Species serve as fundamental units in biology [21]. Species delimitation based on new coalescent methods using multilocus data has been regarded as one of the most exciting developments in systematics, because it tests alternative hypotheses of lineage divergence that allows for gene tree discordance, which often thwarts phylogenetic species identification [22]. Coalescent-based species delimitation has proven to be a powerful tool not only for delimiting and validating species, but also for discovering cryptic species, including cave organisms [23], and is recommended as an important component of integrative taxonomy [24].

In this study, we first validated each *Nesticella* species that had been identified morphologically and further investigated the potential cryptic diversity within these species in a coalescent framework [25]. Moreover, to better understand the evolutionary history of *Nesticella* spiders, we estimated divergence times using both a concatenated gene tree approach and a species tree approach with multilocus sequence data [26]. Our comprehensive sampling of individuals from multiple locations with a wide geographic coverage allowed us to investigate the diversification patterns and evolutionary history of this poorly-known *Nesticella* radiation, the factors that have influenced their diversification, and the origin of *Nesticella* cave spiders in the Yunnan–Guizhou Plateau.

## Methods

### Taxon sampling and sequence analyses

**Figure 1:**
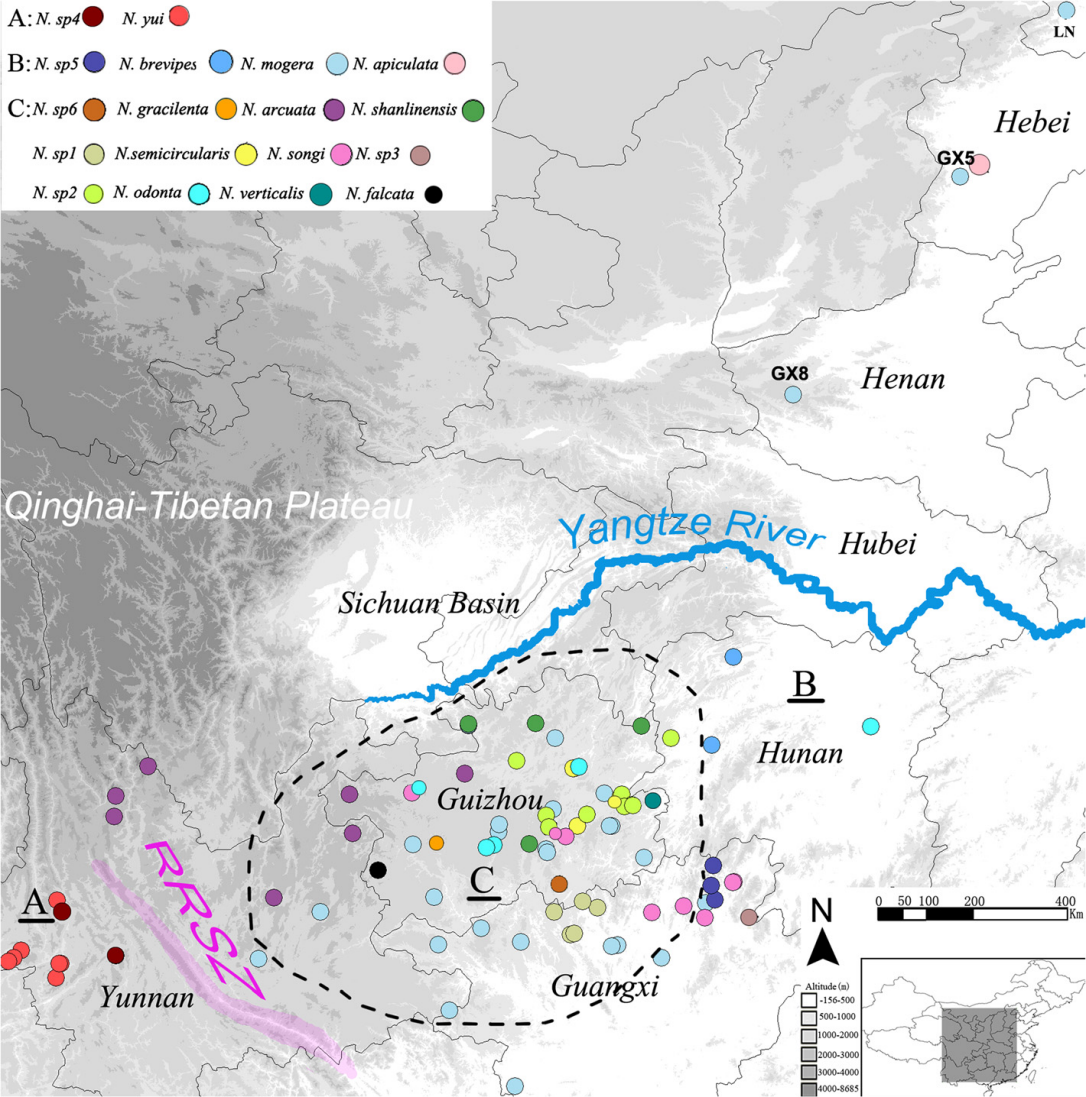
**Sampling sites of *Nesticella *cave spiders in China.** Each species is indicated by a different color, which is consistent through all the figures. RRSZ, Red River Shear Zone. Dotted lines approximately encircle the Yunnan–Guizhou Plateau. The three main clades **(A, B, and C)** are also labeled.

*Nesticella* spiders were collected across the Yunnan–Guizhou Plateau in southern China, and a few samples were collected outside the plateau. A total of 100 *Nesticella* specimens from 100 localities were used in this study (Figure 1). Each population was given an alphanumeric or alphabetic code according to the sampling site, such as SH1, BF1, and EC. All individuals were collected in caves except for one *N. mogera* from a population beneath rocks on the surface near caves. Type species from the sister genus of *Nesticella*, *Nesticus cellulanus* (Nesticidae) and members of the sister family of Nesticidae, Theridiidae (*Theridion* sp.) were sampled as outgroups. Sequences of *Nesticus cellulanus* were downloaded from GenBank (Accessions GU682834, AF124961, and AF005447). Additional file 1 summarizes the taxonomy, locality information, population codes, and sequence accession numbers used in the analyses.

Genomic DNA was extracted from leg or thorax tissue using a standard phenol: chloroform method for one specimen per cave. We sequenced three mitochondrial and three nuclear fragments: the 5′ end of mitochondrial cytochrome c oxidase subunit 1 (*cox1*); the mitochondrial 12S/16S ribosomal RNA (rRNA) genes (including the short tRNA-VAL gene); nuclear histone 3 gene (H3); and fragments of the large (28S, internal fragment) and small (18S, 3′ end) nuclear rRNA. PCR procedures and primers are described in Additional file 2.

Sequences were edited using BioEdit [27]. Protein-coding gene sequences (*cox1* and H3) were aligned in Clustal X v. 2.0 [28] and corrected with visual inspection and translation. The 12S/16S, 18S, and 28S rRNA sequences were aligned in MAFFT (http://mafft.cbrc.jp/alignment/server/) using the L-INS-i strategy to increase accuracy [29]. Highly variable regions of 12S/16S rRNA sequences were parsed using Gblocks [30] with default settings to avoid including unreliable phylogenetic signals. Unique sequences were assigned alphanumeric haplotype codes, and all data were deposited in GenBank under accession numbers KF359001–KF359476 (Additional file 1). Models of DNA sequence evolution for each gene were selected using jModelTest [31] under the Akaike information criterion.

### Phylogenetic analysis

To estimate phylogenetic relationships, Bayesian inference (BI) and maximum likelihood (ML) were implemented for a concatenated dataset of all loci, as well as for concatenated mitochondrial and nuclear sequences separately. Each population was represented by a single individual in this analysis. Maximum likelihood analyses were conducted in RAxML [32] using the GTRGAMMA model for all genes to find the best tree. The GTRGAMMA model in RAxML is recommended over the GTR + Γ + I because the 25 rate categories account for potentially invariant sites. One hundred replicate ML inferences were initiated with a random starting tree and employed the default rapid hill-climbing algorithm. Clade confidence was assessed with 1000 rapid bootstrap replicates. Bayesian inference was performed in MrBayes 3.1.2 [33] using parameters identified by jModelTest. Four Monte Carlo Markov chains (MCMCs) with default heating parameters were performed for 20 million generations to ensure that the average standard deviation of split frequencies was less than 0.01. The Markov chains were sampled every 1000 generations, with the first 25% of sampled trees discarded as burn-in. The 50% majority rule consensus trees with posterior probabilities (PPs) were constructed from the remaining post-burn-in samples.

### Candidate species delimitation

Details of the morphology-based identification procedures are provided in Additional file 2. We identified 12 nominate species and six provisional ones that probably represent novel species; the latter were temporarily named *Nesticella sp1–6*. A paper describing the morphology of these novel species is in preparation (Chen and Li, unpublished).

To validate our morphological inspection, a multilocus coalescent species delimitation analysis was conducted using the program BPP [25,34] with a reversible-jump MCMC method to estimate the posterior distribution for different species delimitation models. We used the multispecies coalescent model implemented in *BEAST [26] to generate a guide tree for the BPP analysis. The nucleotide substitution and molecular clock models were unlinked for all loci, and tree models were unlinked for all nuclear genes but linked for the two mitochondrial genes. We ran two independent analyses for 50 million generations, sampling every 5000 generations, and excluded the first 20% of trees as burn-in. Convergence was assessed by examining likelihood plots through time using TRACER v. 1.4.1 [35]. The *BEAST guide tree was a multilocus species tree needing pre-designated species affiliations. Because we wanted to validate the morphological inspection and test the existence of potentially cryptic species, all the species identified by morphology and deep internodes within species suggested by the phylogenetic tree were treated as operational taxonomic units in *BEAST. We tested a total of 31 operational taxonomic units (Additional file 3). The topology of the guide tree played a critical role in the outcome of the BPP analyses [36]. All nodes tested were highly supported by the phylogenetic analyses, ruling out the plausibility of alternative topologies.

Exploratory analyses using algorithms 0 or 1 with different fine-tuning parameters did not affect speciation probabilities significantly (results not shown), so we only applied algorithm 0 with the parameter ϵ = 5. Each analysis was run at least twice to confirm consistency. Analyses were run for 100,000 generations and sampled every five generations, with a burn-in of 50,000. We evaluated the influence of priors θ (ancestral population size) and τ (root age) by considering three different combinations. We set both θ and τ to a gamma distribution, with (1) G(α, β) ~ G(1,10) for both θ and τ, assuming relatively large ancestral populations and deep divergences; (2) G(2,2000) for both θ and τ, assuming relatively small ancestral populations and shallow divergences among species, favoring conservative models; or (3) G(2,1000) for θ and G(1,10) for τ, assuming small ancestral populations and relatively deep divergences among species. Other divergence time parameters were assigned the Dirichlet prior. Under this approach, the validity of a speciation event is strongly supported by a PP of *P* ≥ 0.95 [36].

### Divergence time estimates

Our divergence time estimates covered both intra- and interspecific levels; therefore, we estimated divergence times first under a gene-tree framework using the concatenated data matrix and second with a multilocus species tree approach. We estimated divergences with the concatenated gene tree approach in BEAST v. 1.7.4 [37]. Substitution models obtained from jModelTest were used for each locus for all 100 populations. An uncorrelated lognormal relaxed clock model was assumed for each partition with a Yule speciation process prior on branching rates.

The dataset was then analyzed using a multispecies coalescent tree model implemented in *BEAST [26]. This method is believed to be more accurate and produces shallower time estimates than concatenated gene-tree based approaches [38], as it implements a full Bayesian inference of the species tree under the multispecies coalescent model. Haplotypes within species follow a coalescent tree model, and relationships among species follow a Yule tree model. Species traits for the *BEAST analysis were defined based on candidate species delimitation. The multi-individual dataset consisted of all DNA fragments for the 100 populations. We unlinked the substitution models and molecular clock for the all-loci and nuclear gene tree models, and linked the two mitochondrial tree models. An uncorrelated lognormal relaxed clock model was assumed for each partition.

For each of these two analyses, four parallel runs of 50 million generations were performed with sampling every 5000 generations, and the first 40% of samples were discarded as burn-in. Log and tree files were combined using LogCombiner, distributed as part of the BEAST package. The final tree was based on 24,000 trees (with 16,000 burn-in trees discarded), and all effective sample size (ESS) values were greater than 200. Posterior probabilities indicated support for nodes. TRACER v. 1.4 was used to determine convergence, measure the ESS of each parameter, and calculate the mean and 95% highest posterior density (HPD) interval for divergence times. The consensus tree was compiled in TreeAnnotator v. 1.4.7 and the chronogram edited in FigTree v. 1.3.1 (http://tree.bio.ed.ac.uk/software/figtree/).

Because of the lack of adequate nesticid fossils, we dated the phylogeny using two paleogeographic events thought to have shaped the biogeography of *Nesticella* spiders. First, the left-lateral strike slip along the Red River Shear Zone, which caused the offshore rift between Indochina and South China, is estimated to have started ca. 17–29 million years ago (Ma), most probably around 22 Ma [39-41]. This zone caused a major geological discontinuity between South China and Indochina [42], and we assumed it caused the divergence between Clade A and all other clades in our species tree. A normally distributed calibration prior was set for this split, with a mean of 22 Ma and standard deviation of 2 Ma. Second, the subdivision of *N. mogera* populations to the south and north of the Yangtze River was attributed to the formation of the Yangtze Gorge (1.8–1.16 Ma) [43]. A normally distributed calibration prior was set for the age of the *N. mogera* clade root, with a mean of 1.5 Ma and standard deviation of 0.18 Ma. The two major lineages of *N. mogera* were designated as species to set the age of the most recent common ancestor of *N. mogera* in BEAUti v. 1.7.4 [37]. We chose normally distributed age priors to place higher probabilities on intermediate dates and lower probabilities on older and younger dates, which was most plausible in our case.

## Results

### Sequence analysis

We successfully sequenced mitochondrial *cox1* and nuclear H3, 28S rRNA, and 18S rRNA for each individual. However, several 12S/16S rRNA sequences, especially for *N. mogera*, could not be recovered because of their high AT content (Additional file 1). High AT content regions frequently yielded ambiguous chromatograms or ceased before terminals. Because mitochondrial sequences were similar among populations of *N. mogera*, we believe that these missing data did not influence our analyses. As a test, we excluded the 12S/16S data, and the resulting phylogeny and the BEAST analyses were not affected (results not shown). The complete concatenated alignment was 3,605 bp long (*cox1*, 623 bp; H3, 304 bp; 18S, 797 bp; 28S, 790 bp; 12S/16S, 1091 bp), of which 2,575 characters were constant, 111 were parsimony-uninformative, and 919 were parsimony-informative. In the 12S/16S alignment, 153 bp were excluded because of alignment ambiguity. The 18S and 28S sequences evolved conservatively, so all positions were reliably aligned and none were excluded. Best-fit models selected by jModelTest were TrN + I + G for *cox1*, TPMuf + I + G for 12S/16S, TPM1 + I for H3, TrN + G for 28S, and TrNef + G for 18S.

### Phylogenetic analysis

**Figure 2:**
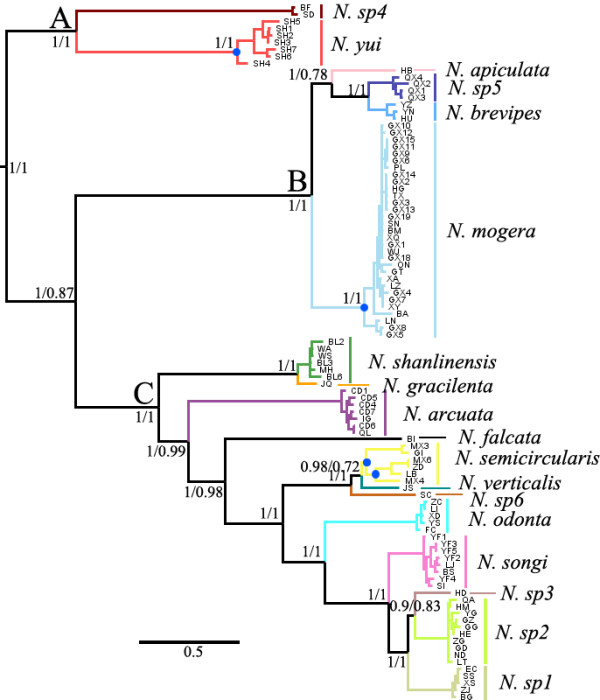
**Phylogenetic tree reconstructed using Bayesian inference based on concatenated data.** Outgroups *Nesticus cellulanus* and *Theridion* sp. were distantly-related to the ingroup and not included in the figure. Numbers beside nodes are posterior probabilities followed by bootstraps values from the maximum likelihood analysis. Blue dots within species *N. yui*, *N. mogera*, and *N. semicircularis* indicate nodes suggested as speciation events by BPP analyses. Clade A, B and C were labeled beside each clade root.

The concatenated gene trees reconstructed by ML were topologically identical to the BI tree. This topology was also consistent with the guide species tree we constructed using *BEAST for the BPP analyses (Additional file 3). Three monophyletic clades, Clades A, B and C, were strongly supported in both analyses (BP = 1, PP = 1) (Figure 2). Clade A branched first, then Clades B and C split. Nodes in the ML and BI trees were consistently supported by BP and PP values. However, the mitochondrial and nuclear gene trees differed in the branching patterns (Additional file 4). The nuclear gene tree supported the topology (A, (B, C)), while the mitochondrial gene tree supported ((A, B), C).

### Candidate species delimitation

The BPP analysis validated all the 18 species identified by morphology (PP = 1 for all) and collapsed most of the internodes within species lineages. The speciation events supported by the BPP analyses are shown in Additional file 3. After exploratory analyses using different algorithms and fine-tuning parameters, we found that these settings did not significantly affect speciation probabilities; however, different combinations of θ and τ resulted in some incongruence. Prior combinations of θ ~ G(1, 10) and τ ~ G(1, 10) showed lower speciation probabilities than did prior combinations with small population sizes and shallow divergence times. BPP found consistent speciation events using all three prior combinations. These results were also consistent across runs, and in all cases, ESS values exceeded 200, indicating convergence of the MCMC chains.

Internodes within *N. mogera*, *N. semicircularis*, and *N. yui* were supported by high speciation probabilities, indicating that these groups may contain cryptic species. Posterior probabilities were high within *N. semicircularis* with all prior combinations (>0.95), but internodes within *N. mogera* and *N. yui* with prior combinations of θ ~ G(1, 10) and τ ~ G(1, 10) collapsed, and were supported as speciation events only with the other two prior combinations (Additional file 3).

### Divergence time estimates

**Figure 3:**
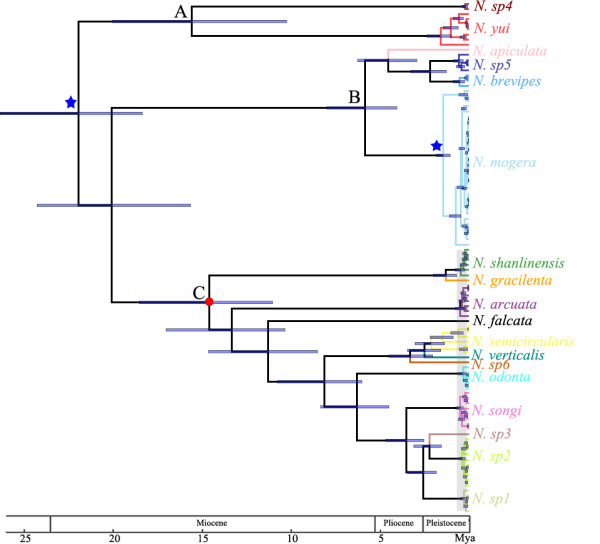
**Chronogram of *Nesticella *spiders.** The chronogram was obtained using BEAST. The geologic time scale is indicated in millions of years below the tree. Blue stars mark the two calibration points. Blue node bars indicate the 95% highest posterior density interval for divergence estimates. The red dot corresponds to the root of the plateau *Nesticella* cave spiders. The grey shaded area indicates the time frame when mass cave colonization events happened. Clade A, B and C were labeled beside each clade root.

Because the species level divergence times estimated in *BEAST (Additional file 5) were only slightly shallower than those in the gene tree approach (Figure 3) as predicted, the following description is only based on the chronogram reconstructed in BEAST.

Calibrated with two geological events, the estimated *cox1* mean substitution rate was 1.68% per million years (MY), equivalent to a divergence rate of 3.36% per MY. This rate was comparable to that estimated by Papadopoulou *et al.*[44], who through an extensive survey of tenebrionid beetles obtained a divergence rate of 3.54% per MY for *cox1*. Our estimated rates for mitochondrial genes were higher than those of Bidegaray-Batista *et al.*[45], probably because of our inclusion of population level comparisons [46]. The estimated substitution rates for H3, 28S, and 18S were 0.066%, 0.16%, and 0.076%, respectively, with 18S and 28S showing high rate heterogeneity among lineages.

The common ancestor of Chinese *Nesticella* was estimated at 21.5 Ma (95% HPD: 18.0–25.7 Ma), when Clade A, distributed in western Yunnan Province, first branched off; it later diverged into two species: *N. sp4* and *N. yui*. Clade B mainly occupied the eastern margin of this plateau and diverged 19.6 Ma (95% HPD: 15.2–24 Ma). Clade C, containing all troglobitic spiders distributed in the main plateau region, formed a tight monophyletic cluster hereafter referred to as the plateau clade. The plateau *Nesticella* clade shared a common ancestor 14.6 Ma (95% HPD: 11.1–18.6 Ma) and comprised 12 species. Most interspecific divergence happened before the Pleistocene, while intraspecific diversification in the plateau and other clades occurred exclusively during the Pleistocene, especially the middle to late Pleistocene.

## Discussion

### Species delimitation

The 18 morphologically-identified species corresponded to well-defined lineages in the phylogenetic tree and were also validated by our coalescent-based species delimitation. Furthermore, the BPP analyses suggested that *N. mogera*, *N. semicircularis*, *and N. yui* as currently defined probably contained cryptic species. Although speciation events were not consistently supported under different prior combinations within *N. mogera* and *N. yui*, we believe that cryptic species probably exist within them because of the deep divergences within the species. Future work to confirm these cryptic species should apply other coalescent-based methods to accommodate different assumptions, such as the general mixed Yule coalescent (GMYC) method [47] and O’Meara’s method [48].

### Phylogenetic analyses

Despite incongruences between the mitochondrial and nuclear gene trees, the concatenated gene tree and the species tree reconstructed with multilocus data strongly supported the same topology separating *Nesticella* into three geographically well-defined clades: A, B, and C. The combined gene tree was dominated by nuclear data, which was not unexpected since Fisher-Reid and Wiens [49] suggested that nuclear genes had lower levels of homoplasy than mitochondrial genes. The relatively rapidly evolving mitochondrial genes supported the monophyly of Clades A plus B, which probably resulted from ancestral polymorphisms and lineage sorting [49]. Overall, our data was sufficient to resolve the phylogenetic relationship among the taxa.

### Evolutionary history of *Nesticella* spiders

We concluded that *Nesticella* cave spiders are an ancient lineage containing young troglobites whose interspecific diversification has been greatly influenced by tectonic movement, but whose intraspecific diversification reflects multiple, recent cave colonizations. These spiders most probably invaded the subterranean habitat during the Pleistocene. Although the Plateau clade (Clade C) was composed exclusively of troglobites, we rejected a scenario of a single troglobite ancestor followed by subterranean dispersal, and instead concluded that *Nesticella* colonized subterranean environments recently and repeatedly.

According to our time estimates, the *Nesticella* lineage is ancient. The chronogram suggested that *Nesticella* spiders originated in the early Miocene (19.6 Ma; Figure 3). During the middle Miocene to late Pliocene, *Nesticella* underwent substantial diversification. Interspecific diversification appears to have been greatly influenced by tectonic movement. The topography of China forms a three-step staircase in which the Yunnan–Guizhou Plateau constitutes the southern part of the second step. The formation of the staircase landform is believed to be closely related to the discontinuous and differential uplift of the Qinghai–Tibetan Plateau, which greatly affected tectonic movements in the Yunnan–Guizhou Plateau, forming mountains and deep valleys and rearranging major river drainages [50,51].

Yunnan–Guizhou Plateau *Nesticella* cave spiders formed a well-supported clade whose common ancestor separated from its relatives 14.6 Ma, when eastern Tibet had initiated a rapid uplift [52,53]. The independent evolutionary history of the plateau clade (Clade C) indicated a strong geology-induced impact on biogeography. The early members of the plateau clade were fragmented by geographic barriers formed by the intense tectonic movement during this uplift [54]. The early split of species *N. gracilenta*, *N. shanlinensis*, and *N. arcuata* in the northern plateau area from the other species in the central and southern areas also indicated that the diversification of the plateau group has been greatly influenced by the northwest to southeast sequential uplift of the Yunnan–Guizhou Plateau. All well-supported deep clades were restricted to geographically distinct regions, a pattern common to other studies [16,17].

We are confident there were no unsampled epigean species belonging to the Plateau Clade C for two reasons: a) over several years, we sampled intensively from both potential surface habitats and caves and b) there are no reports of epigean *Nesticella* species except for *N. mogera*, which is unrelated to this clade. Sampling in other areas was less intensive, and we cannot exclude the possibility of unsampled epigean populations or species in Clades A and B. Overall, the Plateau *Nesticella* clade was composed exclusively of troglobites.

We concluded that a single cave colonization scenario was not likely for plateau cave *Nesticella*. Although all plateau cave spiders formed a well-supported monophyletic group containing no epigean species, suggesting that all the troglobites shared a single common subterranean ancestor that subsequently diversified, the scenario that *Nesticella* spiders colonized caves in the early Miocene and subsequently dispersed over a large geographic area is highly improbable; the lineage divergence during the Pleistocene denied the possibility of an ancient cave colonization. If *Nesticella* colonized caves in ancient times, we could expect that the lineages diverged in response to the geological movement in this plateau, such as the formation of geological barriers and the recurring cave formation-to-dilapidation process; instead, we observed a burst of divergence during the Pleistocene, which most probably resulted from the dramatic climate changes during that time. Surface climate change would not have such notable effects on lineage divergence if *Nesticella* had already adapted to the cave environment and there was no indication that geological movement had intensified during the Pleistocene.

The burst of diversification during the Pleistocene was most probably affected by climate fluctuations. Pleistocene glaciations significantly impacted the distribution and diversity of subterranean fauna [55], including cave organisms [56,57]. During glacial advances, the unfavorable cold and dry climate forced epigean species to take refuge in the warm and humid habitats of caves [58]. Most of this diversification happened during the middle to late Pleistocene (Figure 3), consistent with the well-known onset of prominent glaciation cycles around 0.8 to 1 Ma [59]. One lineage (*N. semicircularis*) diverged in the early Pleistocene, indicating that these spiders might have responded to climate fluctuations earlier than other lineages.

Because of the absence of epigean species in the plateau lineage, we could not determine the origin of these cave spiders. Similarly, neither the climate-relict nor the adaptive-shift hypotheses could be completely ruled out. Regardless of which hypothesis is more plausible, the fact that all *Nesticella* cave spider populations inhabited isolated caves supported their relict status. Moreover, as no epigean species in the plateau clade were sampled, we still cannot determine which nodes in the tree corresponded to the surface-to-cave transition events. To find the exact transition point, one first needs comprehensive sampling of all the existing populations (including epigean populations) within this clade and second to rule out the possibility of vertical movement, which is the migration of animals from older caves to the caves they inhabit now. Unfortunately, exhaustive sampling is impossible in empirical work, especially when epigean populations have already gone extinct, and vertical movement is also a possibility, as some studies have suggested that old troglobites inhabited young caves through vertical movement [16,17,60]. Nevertheless, with extensive sampling across the plateau and combining phylogenetic trees with time estimates and geological events, we could determine whether troglobites belonging to the monophyletic cave lineage were ancient or young.

## Conclusions

This study evaluated the species diversity and evolutionary history of the poorly studied *Nesticella* spiders. Using a multilocus species tree method for divergence time estimates, we found that the plateau *Nesticella* cave spiders constituted a monophyletic clade that originated in the early Miocene. Although all plateau cave spiders formed a well-supported monophyletic group lacking epigean species, we rejected an ancient colonization scenario. We concluded that the interspecific divergence of *Nesticella* was influenced by ancient geological movements, and more recent divergence probably reflects their cave colonization history, which was a response to Pleistocene glaciations. Comprehensively sampled studies of cave organisms have usually focused on species delimitation and cryptic diversity. Our work showed that comprehensive sampling also offers a better understanding of the evolutionary history of cave organisms. With dense sampling centered in the Yunnan–Guizhou Plateau, we acquired 18 candidate species, among which six were not previously identified. Using newly developed coalescent-based species delimitation methods, we validated our morphological identifications and further found potentially cryptic lineages requiring further study. The Yunnan–Guizhou Plateau relict cave spiders are important components of the local biodiversity, which is vulnerable both evolutionarily and ecologically. The existence of cryptic species and the relict status of these troglobites highlight the importance of cave conservation to protect interspecific and intraspecific diversity.

## Competing interests

Both authors declare that they have no competing interests.

## Authors’ contributions

SL designed the study. YZ performed molecular labwork, conducted analyses and wrote the manuscript. Both authors read and approved the final manuscript.

## Supplementary Material

Additional file 1 **Information S1.** Details of the 100 *Nesticella* and 2 outgroup populations’ codes, localities, coordinate information and GenBank accession numbers; individual populations were named alphanumerically and undescribed species were given provisional alphabetic names. Missing data were indicated by “–”. Click here for file

Additional file 2 Material and methods. Click here for file

Additional file 3 **The figure above was the detailed results of the coalescent based species delimitation methods and below was photos of each species.** The species guide tree were constructed using *Beast. Tips of the tree, named by species names followed by an underscore and population codes, were operational taxonomic units testing in BPP. Posterior probabilities were shown below the nodes with prior combinations of θ ~ G(1, 10) and τ ~ G(1, 10), θ ~ G(2, 2000) and τ ~ G(2, 2000) and θ ~ G(2, 2000) and τ ~ G(1, 10). PP values lower than 0.95 across all prior combinations were omitted. Posterior probabilities of the guide species tree were shown above the nodes. Clade colors for each species were consistent with the sampling map. There is no photo for *N. sp3* because we only sample one specimen of it and this specimen was destroyed during DNA extraction. Click here for file

Additional file 4 **The Bayesian gene tree reconstructed using mitochondrial (left) and nuclear (right) sequences.** Outgroups *Nesticus cellulanus* and *Theridion* sp. were distantly related with the ingroup and not included. Numbers above nodes are posterior probabilities followed by maximum likelihood bootstraps and letters below nodes are the three main clade names. Colors of braches correspond to colors in Figure 1. Click here for file

Additional file 5: **Chronogram of *Nesticella* age divergences with 95% confidence intervals (blue bars).** Numbers besides nodes were node ages. Click here for file
