# Supplementary material for: Ancient lineage, young troglobites: recent colonization of caves by Nesticella spiders
Source: BMC Evol Biol. 2013 Sep 4;13:183. doi: 10.1186/1471-2148-13-183 (PMC3766682; doi:10.1186/1471-2148-13-183)
Supplement: Additional file 1 — Information S1. Details of the 100 Nesticella and 2 outgroup populations’ codes, localities, coordinate information and GenBank accession numbers; individual populations were named alphanumerically and undescribed species were given provisional alphabetic names. Missing data were indicated by “–”. [file 1471-2148-13-183-S1.docx]

|  |  | Sampling site | | GenBank accession number | | | | |  |
| --- | --- | --- | --- | --- | --- | --- | --- | --- | --- |
| Species | Population code | Locality | Coordinate | *cox1* | 12S/16S | H3 | 28s | 18s |  |
| *Nesticella sp1* | EC | Encun Cave,Nandan County, Guangxi Province, China | N25º04.159´E107º36.195´ | KF359002 | KF359404 | KF359120 | KF359304 | KF359203 | |
| *Nesticella sp1* | SS | Shoushui Cave, Hechi City, Guangxi Province, China | N24º41.229´E107º52.609´ | KF359004 | KF359405 | KF359122 | KF359306 | KF359205 | |
| *Nesticella sp1* | XS | Xiaoshui Cave, Huanjiang County, Guangxi Province, China | N25º14.950´E108º04.70´ | KF359003 | KF359406 | KF359121 | KF359305 | KF359204 | |
| *Nesticella sp1* | BG | Pusa Cave, Libo County, Guizhou Province, China | N25º14.950´E108º04.70´ | KF359005 | KF359407 | KF359123 | KF359307 | KF359206 | |
| *Nesticella sp1* | ZJ | Zhenzhu Cave, Hechi City, Guangxi Province, China | No detailed location data | KF359006 | - | KF359124 | KF359308 | KF359207 | |
| *Nesticella sp2* | YG | Yangzi Cave,Fuquan City,Guizhou Province, China | N26º42.275´E107º27.96´ | KF359013 | KF359409 | KF359117 | KF359315 | KF359214 | |
| *Nesticella sp2* | HM | Hama Cave, Fenghuang County, Hunan Province, China | N27º59.5´ E109 º33.8´ | KF359014 | KF359408 | KF359113 | KF359316 | KF359215 | |
| *Nesticella sp2* | HE | Hei Cave, Zunyi City, Guizhou Province,China | N27º36.759´E106º58.08´ | KF359008 | KF359410 | KF359114 | KF359310 | KF359209 | |
| *Nesticella sp2* | QA | Qiliang Cave, Fenghuang County, Hunan Province, China | No detailed location data | KF359007 | KF359411 | KF359125 | KF359309 | KF359208 | |
| *Nesticella sp2* | GD | Guyang Cave, Taijiang County, Kaili City, Guizhou Province, China | N26º42.85´E108º08.945´ | KF359010 | KF359412 | KF359110 | KF359312 | KF359211 | |
| *Nesticella sp2* | ZG | Zhangjiawan Cave, Zhenyuan County, Kaili City, Guizhou Province, China | N27º1.8´E108º 26.6´ | KF359011 | KF359413 | KF359111 | KF359313 | KF359212 | |
| *Nesticella sp2* | LT | Long Cave, Sansui County, Kaili City, Guizhou Province, China | N26º50.846´E108º47.18´ | KF359015 | KF359414 | KF359118 | KF359317 | KF359216 | |
| *Nesticella sp2* | ND | Niu Cave, Jianhe County, Kaili City, Guizhou Province, China | N26º51.718´E108º55.70´ | KF359012 | KF359415 | KF359112 | KF359314 | KF359213 | |
| *Nesticella sp2* | GZ | Guazhutou cave, Majiang County, Kaili City, Guizhou Province, China | N26º30.257´E107º 30.94´ | KF359009 | KF359416 | KF359115 | KF359311 | KF359210 | |
| *Nesticella sp2* | GG | Gang Cave, Jianhe County, Kaili City, Guizhou Province, China | N26º52.320´E108º54.41´ | KF359016 | KF359417 | KF359116 | KF359318 | KF359217 | |
| *Nesticella sp3* | HD | Hou Cave, Gongcheng County, Guangxi Province, China | N24º58.045'E110º52.867' | KF359017 | KF359418 | KF359177 | KF359319 | KF359218 | |
| *Nesticella songi* | YF1 | Yongfu Cave, Yongfu County, Guangxi Province, China | N24º58.128'E110º08.362' | KF359020 | KF359419 | KF359104 | KF359322 | KF359221 | |
| *Nesticella songi* | YF2 | Unnamed Cave, Rongshui County, Guangxi Province, China | N25º03.604'E109º14.680' | KF359018 | KF359420 | KF359102 | KF359320 | KF359219 | |
| *Nesticella songi* | YF3 | Dayan Cave, Xing’an County, Guangxi Province, China | N25º34.263'E110º36.594' | KF359024 | - | KF359108 | KF359326 | KF359225 | |
| *Nesticella songi* | YF4 | Baishou Cave, Yongfu County, Guangxi Province, China | N25º10.028'E109º46.696' | KF359025 | - | KF359109 | KF359327 | KF359226 | |
| *Nesticella songi* | YF5 | Ruyan Cave, Xing’an County, Guangxi Province, China | N25º34.437'E110º37.237' | KF359019 | KF359421 | KF359103 | KF359321 | KF359220 | |
| *Nesticella songi* | BS | Shuibashui Cave, Libo County, Guizhou Province,China | N27º04.935´E105º12.809´ | KF359022 | KF359422 | KF359106 | KF359324 | KF359223 | |
| *Nesticella songi* | SI | Shuilong Cave, Danzhai County, Kaili City, Guizhou Province, China | N26º20.615´E107º48.02´ | KF359023 | KF359423 | KF359105 | KF359325 | KF359224 | |
| *Nesticella songi* | LJ | Unnamed Cave, Majiang County, Kaili City, Guizhou Province, China | N26º28.646´E107º32.49´ | KF359021 | KF359424 | KF359105 | KF359323 | KF359222 | |
| *Nesticella* *arcuata* | CD1 | Chuan Cave, Jinsha County, Guizhou Province,China | N27º23.797´E106º06.104´ | KF359026 | KF359425 | KF359126 | KF359328 | KF359227 | |
| *Nesticella arcuata* | CD4 | Bianfu Cave, Lijiang City, Yunnan Province,China | N27º01.357'E100º13.825' | KF359028 | KF359426 | KF359128 | KF359330 | KF359229 | |
| *Nesticella arcuata* | CD5 | Biyigu Cave, Lijiang City, Yunnan Province,China | N27º31.244'E100º46.306' | KF359027 | KF359427 | KF359127 | KF359329 | KF359228 | |
| *Nesticella arcuata* | CD6 | Qingxuan Cave, Dali City,  Yunnan Province,China | N26º40.449'E100º12.443' | KF359029 | KF359428 | KF359129 | KF359331 | KF359230 | |
| *Nesticella arcuata* | CD7 | Long Cave, Qujing City,  Yunnan Province,China | N26º23.412'E104º12.744' | KF359030 | KF359429 | KF359130 | KF359332 | KF359231 | |
| *Nesticella arcuata* | QL | Qinglong Cave, Songming County, Yunnan Province,China | N25º18.355´E102º53.304´ | KF359032 | KF359430 | KF359132 | KF359334 | KF359233 | |
| *Nesticella arcuata* | IG | Gui Cave, Weining County, Bijie City, Guizhou province, China | N27º03.382´E104º09.70´ | KF359031 | KF359431 | KF359131 | KF359333 | KF359232 | |
| *Nesticella* *falcata* | BI | Biyun Cave, Pan County, Guizhou Province, China | N25º46.509´E104º38.263´ | KF359033 | KF359432 | KF359133 | KF359335 | KF359234 | |
| *Nesticella sp4* | BF | Bianfu Cave, Lincang City, Yunnan Province, China | N24º19.862'E100º14.001' | KF359034 | KF359433 | KF359199 | KF359336 | KF359235 | |
| *Nesticella sp4* | SD | Qingshuigou Cave, Baoshan City, Yunnan Province, China | N25º04.445´E99º20.140´ | KF359035 | KF359434 | KF359200 | KF359337 | KF359236 | |
| *Nesticella yui* | SH1 | Shihuan Cave, Tengchong County, Yunnan Province, China | N24º25.464'E98º38.304' | KF359040 | KF359435 | KF359197 | KF359342 | KF359241 | |
| *Nesticella yui* | SH2 | Xianfo Cave, Luxi City, Yunnan Province, China | N24º19.971'E98º30.943' | KF359041 | KF359436 | KF359134 | KF359343 | KF359242 | |
| *Nesticella yui* | SH3 | Sanxian Cave, Luxi City, Yunnan Province, China | N24º13.929'E98º25.563' | KF359042 | KF359437 | KF359135 | KF359344 | KF359243 | |
| *Nesticella yui* | SH4 | Xianren Cave, Lincang City, Yunnan Province, China | N24º12.099'E99º18.607' | KF359036 | KF359438 | KF359193 | KF359338 | KF359237 | |
| *Nesticella yui* | SH5 | Guanyin Cave, Lincang City, Yunnan Province, China | N23º57.729'E99º13.918' | KF359037 | KF359439 | KF359194 | KF359339 | KF359238 | |
| *Nesticella yui* | SH6 | Riyue Cave, Lincang City, Yunnan Province, China | N24º11.888'E99º16.615' | KF359038 | KF359440 | KF359195 | KF359340 | KF359239 | |
| *Nesticella yui* | SH7 | Shihua Cave, Baoshan City, Yunnan Province, China | N25º15.846'E99º14.715' | KF359039 | KF359441 | KF359196 | KF359341 | KF359240 | |
| *Nesticella brevipes* | HU | Huanglong Cave, Zhangjiajie City, Hunan Province, China | No detailed location data | KF359043 | KF359443 | KF359136 | KF359345 | KF359244 | |
| *Nesticella* *brevipes* | YN | Yanzi Cave, Chenxi County, Hunan Province, China | No detailed location data | KF359044 | KF359442 | KF359137 | KF359346 | KF359245 | |
| *Nesticella* *brevipes* | YZ | Yan Cave, Chenxi County, Hunan Province, China | No detailed location data | KF359045 | KF359444 | KF359138 | KF359347 | KF359246 | |
| *Nesticella sp5* | QX1 | Qixing Cave, Guilin City, Guangxi Province, China | N25º16.33'E110º18.25' | KF359046 | KF359445 | KF359139 | KF359348 | KF359247 | |
| *Nesticella sp5* | QX2 | Dushu Cave, Hezhou City, Guangxi Province, China | N25º51.095'E111º16.705' | KF359047 | KF359446 | KF359140 | KF359349 | KF359248 | |
| *Nesticella sp5* | QX3 | Ruyan Cave, Xing’an County, Guangxi Province, China | N25º34.437'E110º37.237' | KF359048 | KF359447 | KF359141 | KF359350 | KF359249 | |
| *Nesticella sp5* | QX4 | Yanbei Cave, Guilin City, Guangxi Province, China | N25º30.776'E110º14.607' | KF359049 | - | KF359142 | KF359351 | KF359250 | |
| *Nesticella* *apiculata* | HB | Huiyinbi , Zanhuang County, Hebei Province, China | N37º26.4'E114 º1.8' | KF359050 | KF359448 | KF359143 | - | KF359251 | |
| *Nesticella* *gracilenta* | JQ | Jinqian Cave, Puding County, Guizhou Province, China | N26º14.009´E105º37.843´ | KF359051 | KF359449 | KF359144 | KF359352 | KF359252 | |
| *Nesticella* *shanlinensis* | BL2 | Guanlong Cave, Xishui County, Guizhou Province, China | N28º13.968´E106º09.96´ | KF359053 | - | KF359146 | KF359354 | KF359254 | |
| *Nesticella shanlinensis* | BL3 | Hejiao Cave, Suiyang County, Guizhou Province, China | N28º14.704´E107º17.30´ | KF359054 | - | KF359147 | KF359355 | KF359255 | |
| *Nesticella shanlinensis* | WS | Woshuida Cave, Guiding County, Guizhou Province, China | N26 º13.2´E107 º108´ | KF359096 | KF359450 | KF359173 | KF359402 | KF359297 | |
| *Nesticella shanlinensis* | MH | Mahuang Cave, Suiyang County, Guizhou Province, China | N28º14.611´E107º17.370´ | KF359055 | KF359451 | KF359148 | KF359356 | KF359256 | |
| *Nesticella shanlinensis* | BL6 | Shanlin Cave, Suiyang County, Guizhou Province, China | N28º14.643´E107º17.28´ | KF359052 | KF359452 | KF359145 | KF359353 | KF359253 | |
| *Nesticella* *shanlinensis* | WA | Mawan Cave, Suiyang County, Guizhou Province, China | N28º11.87´E107 º4.297´ | KF359056 | KF359453 | KF359149 | KF359357 | KF359257 | |
| *Nesticella sp6* | SC | Sanfenhe Cave, Libo County, Guizhou Province, China | N25º32´E107º42´ | KF359057 | KF359454 | KF359150 | KF359358 | KF359258 | |
| *Nesticella* *verticalis* | JS | Jinshanrong Cave, Tianzhu County, Guizhou Province, China | N26º57.669´E109º12.37´ | KF359058 | KF359455 | KF359151 | KF359359 | KF359259 | |
| *Nesticella* *semicircularis* | MX3 | Xiao Cave, Sansui County, Guizhou Province, China | No detailed location data | KF359059 | - | KF359152 | KF359360 | KF359260 | |
| *Nesticella semicircularis* | GI | Gui Cave, Sansui County, Guizhou Province, China | N27º01.241´E108º42.71´ | KF359060 | KF359456 | KF359153 | KF359361 | KF359261 | |
| *Nesticella semicircularis* | MX4 | Panzi Cave, Kaili City, Guizhou Province, China | N26º30.85´E107º59.648´ | KF359061 | KF359457 | KF359154 | KF359362 | KF359262 | |
| *Nesticella semicircularis* | MX6 | Hebian Cave, Kaili City, Guizhou Province, China | N27º29.072´E107º55.74´ | KF359098 | KF359458 | KF359175 | KF359398 | KF359299 | |
| *Nesticella semicircularis* | LB | Lubian Cave, Kaili City, Guizhou Province, China | N27º29.185´E107º55.417´ | KF359097 | KF359459 | KF359174 | KF359397 | KF359298 | |
| *Nesticella semicircularis* | ZD | Zuigeda Cave, Kaili City, Guizhou Province, China | N27º29.185´E107º55.41´ | KF359099 | KF359460 | KF359176 | KF359399 | KF359300 | |
| *Nesticella odonata* | ZC | Zhichang Cave, Changshun County, Guizhou Province, China | N26º11.750´E106º35.230´ | KF359091 | - | KF359168 | KF359392 | KF359292 | |
| *Nesticella odonata* | YS | Yuelu Mountain, Changsha City, Hunan Province, China | N28º11.4´E112º55.77´ | KF359092 | KF359461 | KF359169 | KF359393 | KF359293 | |
| *Nesticella odonata* | LI | Liuguan Cave, Changshun County, Guizhou Province, China | N26º9.148´E106º27.722´ | KF359093 | KF359462 | KF359170 | KF359394 | KF359294 | |
| *Nesticella odonata* | XD | Xiao Cave, Bijie City, Guizhou Province, China | N27º6.639´E105º14.539´ | KF359094 | - | KF359171 | KF359395 | KF359295 | |
| *Nesticella odonata* | FC | Fengchen Cave, Tongren City, Guizhou Province, China | N27º30.876´E108º01.32´ | KF359095 | - | KF359172 | KF359396 | KF359296 | |
| *Nesticella mogera* | GX1 | Guixian Cave, Xishui County, Guizhou Province, China | N28º12.689´E106º09.6´ | KF359062 | - | KF359155 | KF359363 | KF359263 | |
| *Nesticella mogera* | GX2 | Qingtangwan Cave, Sinan County, Guizhou Province, China | No detailed location data | KF359063 | - | KF359156 | KF359364 | KF359264 | |
| *Nesticella mogera* | GX3 | Xianren Cave, Fuquan City, Guizhou Province, China | N26º47.992´E107º35.112´ | KF359064 | - | KF359157 | KF359365 | KF359265 | |
| *Nesticella mogera* | GX4 | Wolong Cave, Haikou City, Hainan Province, China | No detailed location data | KF359065 | - | KF359158 | KF359366 | KF359266 | |
| *Nesticella mogera* | GX5 | Baiyun Cave, Lincheng County, Hebei Province, China | N37°27.35'E114°25.92' | KF359066 | - | KF359159 | KF359367 | KF359267 | |
| *Nesticella mogera* | GX6 | Laohu Cave, Liuzhou City, Guangxi Province, China | N4º17.826'E109º24.168' | KF359067 | KF359463 | KF359160 | KF359368 | KF359268 | |
| *Nesticella mogera* | GX7 | Tiexirong Cave, Kaili City, Guizhou Province, China | N27°04´E108°27´ | KF359068 | KF359464 | KF359201 | KF359368 | KF359269 | |
| *Nesticella mogera* | GX8 | Jiguan Cave, Luoyang City, Henan Province, China | N33°46.74'E111°37.23' | KF359069 | - | KF359161 | KF359370 | KF359270 | |
| *Nesticella mogera* | GX9 | Bailong Cave, Yizhou City, Guangxi Province, China | N24º30.322'E108º39.89' | KF359070 | KF359465 | KF359162 | KF359371 | KF359271 | |
| *Nesticella mogera* | GX10 | Lingui County, Guangxi Province, China | N25º13.03'E110º9.293' | KF359071 | - | KF359163 | KF359372 | KF359272 | |
| *Nesticella mogera* | GX11 | Xiannv Cave, Yizhou City, Guangxi Province, China | N24º29.32'E 108º34.21' | KF359072 | KF359466 | KF359164 | KF359372 | KF359273 | |
| *Nesticella mogera* | GX12 | Unnamed Cave, Rongshui County, Guangxi Province, China | N25º03.6'E 109º14.68' | KF359073 | KF359467 | KF359198 | KF359374 | KF359274 | |
| *Nesticella mogera* | GX13 | Xiniu Cave, Anlong County, Guizhou Province, China | N25º18.88´E105º35.29´ | KF359074 | - | KF359180 | KF359375 | KF359275 | |
| *Nesticella mogera* | GX14 | Qingshan Cave, Qujing City, Yunnan Province, China | N25º4.12'E103º40.28' | KF359075 | - | KF359181 | KF359382 | KF359276 | |
| *Nesticella mogera* | GX15 | Longma Cave, Yuxi City, Yunnan Province, China | N24º28.62'E102º38.18' | KF359076 | KF359468 | KF359182 | KF359376 | KF359277 | |
| *Nesticella mogera* | GT | Gantuo Cave, Napo County, Guangxi Province, China | N23º24.915´E105º50.42´ | KF359077 | KF359469 | KF359183 | KF359377 | KF359278 | |
| *Nesticella mogera* | TX | Tiexice Cave, Zhenyuan County, Guizhou Province, China | N27º29.07´E107º55.73´ | KF359078 | - | KF359184 | KF359378 | KF359279 | |
| *Nesticella mogera* | GX18 | Yutang Cave, Guiyang City, Guizhou Province, China | N26º25.24´E106º39.96´ | KF359088 | - | KF359165 | KF359389 | KF359289 | |
| *Nesticella mogera* | GX19 | Gui Cave, Xishui County, Guizhou Province, China | N28º12.78´E106º09.82´ | KF359089 | - | KF359166 | KF359390 | KF359290 | |
| *Nesticella mogera* | BA | Bailong Cave, Guiyang City, Guizhou Province, China | N:26º32.5´E:106º40.2´ | KF359090 | KF359470 | KF359167 | KF359391 | KF359291 | |
| *Nesticella mogera* | XA | Xi’an Cave, Yizhou County, Guangxi Province, China | N24º33.94´E107º2.46´ | KF359079 | KF359471 | KF359192 | KF359378 | KF359280 | |
| *Nesticella mogera* | LZ | Laizi Cave, Meitan County, Guizhou Province, China | N27º59.4´E107 º37.09´ | KF359080 | KF359472 | KF359202 | KF359380 | KF359281 | |
| *Nesticella mogera* | LN | Changping County, Beijing City, China | N40º15.409´E116º13.204´ | KF359081 | KF359473 | KF359190 | KF359381 | KF359282 | |
| *Nesticella mogera* | BM | Baima Cave,Chongzuo City, Guizhou Province, China | N22º13.552',E106º94.459' | KF359083 | - | KF359185 | KF359384 | KF359284 | |
| *Nesticella mogera* | XY | Xiayan Cave, Baise City, Guizhou Province, China | N4º79.306',E 106º37.218' | KF359085 | - | KF359187 | KF359386 | KF359286 | |
| *Nesticella mogera* | PL | Pulian Cave, Baise City, Guizhou Province, China | N24º51.656',E105º65.948' | KF359082 | KF359474 | KF359191 | KF359383 | KF359283 | |
| *Nesticella mogera* | HG | Houdiao Cave, Jianghe County, Kaili City, Guizhou Province, China | N26º51.851´E108º56.69´ | KF359087 | - | KF359189 | KF359388 | KF359288 | |
| *Nesticella mogera* | ON | Long Cave, Danzhai County, Kaili City, Guizhou Province, China | N26º12.528´E107º46.65´ | KF359084 | KF359476 | KF359186 | KF359385 | KF359285 | |
| *Nesticella mogera* | SN | Shenxian Cave, Jianghe County, Kaili City, Guizhou Province, China | N26º52.205´E108º53.64´ | KF359086 | -- | KF359188 | KF359387 | KF359287 | |
| *Nesticella mogera* | XQ | Unnamed Cave, Danzhai County, Kaili City, Guizhou Province, China | N26º07.653´E107º48.26´ | KF359100 | KF359475 | KF359178 | KF359400 | KF359301 | |
| *Nesticella mogera* | WJ | Wanjian Cave, Liupanshui City, Guizhou Province |  | KF359101 | - | KF359179 | KF359401 | KF359302 | |
| *Theridion* sp*.* |  | Long Cave, Qujing City, Yunnan Province, China | N26º23.412'E104º12.744' | KF359001 | - | KF359119 | KF359403 | KF359303 | |
| *Nesticus cellulanus* |  |  |  | GU682834 | - | - | AF124961 | AF005447 | |

Information S1. Details of the 100 *Nesticella* and 2 outgroup populations’ codes, localities, coordinate information and GenBank accession numbers; individual populations were named alphanumerically and undescribed species were given provisional alphabetic names. Missing data were indicated by “–”.
