# Supplementary material for: Ancient lineage, young troglobites: recent colonization of caves by Nesticella spiders
Source: BMC Evol Biol. 2013 Sep 4;13:183. doi: 10.1186/1471-2148-13-183 (PMC3766682; doi:10.1186/1471-2148-13-183)
Supplement: Additional file 2 — Material and methods. [file 1471-2148-13-183-S2.doc]

**Material and Methods**

**PCR precodues**

PCR reactions were conducted in an Eppendorf thermal cycler (Hamburg, Germany) as follows: 5 min initial denaturation at 94°C; followed by 35 cycles of denaturation at 94°C for 30 s, annealing at 45–50°C for 30 s, and extension at 72°C for 30 s; with a final 5 min extension at 72°C. Purified PCR products were sequenced in both directions on an ABI 377 sequences (Applied Biosystems, Foster City, CA, USA) with the BigDye terminator cycle sequencing ready reaction kit (Applied Biosystems). Primers used in the PCR reactions were listed in the following table.

| *primer* | *gene* | *sequence* | *reference* |
| --- | --- | --- | --- |
| LCO1490 | *cox*1 | 5′-GGTCAACAAATCATCATAAAGATATTGG-3′ | (Folmer et al. 1994) |
| Chelicerate reverse1 | *cox1* | 5′-TACTCTACTAATCATAAAGACATTGG-3′ | (Barrett and Hebert 2005) |
| NES1216F | *rrnl+trnV+rrnS* | 5′-CATTTAATMTYACTAGAGGAACTTGT-3′ | This study |
| NES1216R | *rrnl+trnV+rrnS* | 5′-AATTAATTCAACATCGAGGTCGTAT-3′ | This study |
| H3aF | H3 | 5′-ATGGCTCGTACCAAGCAGACVGC-3′ | (Colgan et al. 1998) |
| H3aR | H3 | 5′-ATATCCTTRGGCATRATRGTGAC-3′ | (Colgan et al. 1998) |
| 28sa | 28s | 5′-GACCCGTCTTGAAACACGGA-3′ | (Rix et al. 2008) |
| LSUR | 28s | 5′-GCTACTACCACCAAGATCTGCA-3′ | (Rix et al. 2008) |
| 5F | 18s | 5′-GCGAAAGCATTTGCCAAGAA-3′ | (Giribet et al. 1999) |
| 9R | 18s | 5′-GATCCTTCCGCAGGTTCACCTAC-3′ | (Giribet et al. 1999) |

**Candidate species delimitation**

Species identification of spiders depends on the structure of copulatory organs including male palps and female epigyna, which function on a “lock and key” principle. Each species of spider have its unique palpal and epigynal structure, which could only be found in mature specimens. We examined the morphology of our collected specimens. Male palps and female epigyna were compared with those of described taxa and the specimens were identified to species when possible (Figure 2).

**Reference**

1. Folmer O, Black M, Hoeh W, Lutz R, Vrijenhoek R: **DNA primers for amplification of mitochondrial cytochrome c oxidase subunit I from diverse metazoan invertebrates.** Mol Mar Biol Biotechnol 1994, **3**(5)**:**294-299.

2. Barrett RDH, Hebert PDN: **Identifying spiders through DNA barcodes.** Can J Zool 2005, **83:**481-491.

3. Colgan DJ, McLauchlan A, Wilson GDF, Livingston SP, Edgecombe GD, Macaranas J, Cassis G, Gray MR: **Histone H3 and U2 snRNA DNA sequences and arthropod molecular evolution.** Austn J Zool 1998, **46**(5)**:**419-437.

4. Rix MS, Harvey JD: **RobertsMolecular phylogenetics of the spider family Micropholcommatidae (Arachnida: Araneae) using nuclear rRNA genes (18S and 28S)** *Mol Phylogenet Evol* 2008, **46**:1031–1048.

5. Giribet G,Carranza S,Riutort M,BaguñàJ, Ribera C: **Internal phylogeny of the Chilopoda (Myriapoda, Arthropoda) using complete 18S rDNA and partial 28S rDNA sequences** Phil Trans R Soc Lond 1999, **354:**215–222.
