# Supplementary material for: Ancient lineage, young troglobites: recent colonization of caves by Nesticella spiders
Source: BMC Evol Biol. 2013 Sep 4;13:183. doi: 10.1186/1471-2148-13-183 (PMC3766682; doi:10.1186/1471-2148-13-183)
Supplement: Additional file 3 — The figure above was the detailed results of the coalescent based species delimitation methods and below was photos of each species. The species guide tree were constructed using *Beast. Tips of the tree, named by species names followed by an underscore and population codes, were operational taxonomic units testing in BPP. Posterior probabilities were shown below the nodes with prior combinations of θ ~ G(1, 10) and τ ~ G(1, 10), θ ~ G(2, 2000) and τ ~ G(2, 2000) and θ ~ G(2, 2000) and τ ~ G(1, 10). PP values lower than 0.95 across all prior combinations were omitted. Posterior probabilities of the guide species tree were shown above the nodes. Clade colors for each species were consistent with the sampling map. There is no photo for N. sp3 because we only sample one specimen of it and this specimen was destroyed during DNA extraction. [file 1471-2148-13-183-S3.doc]

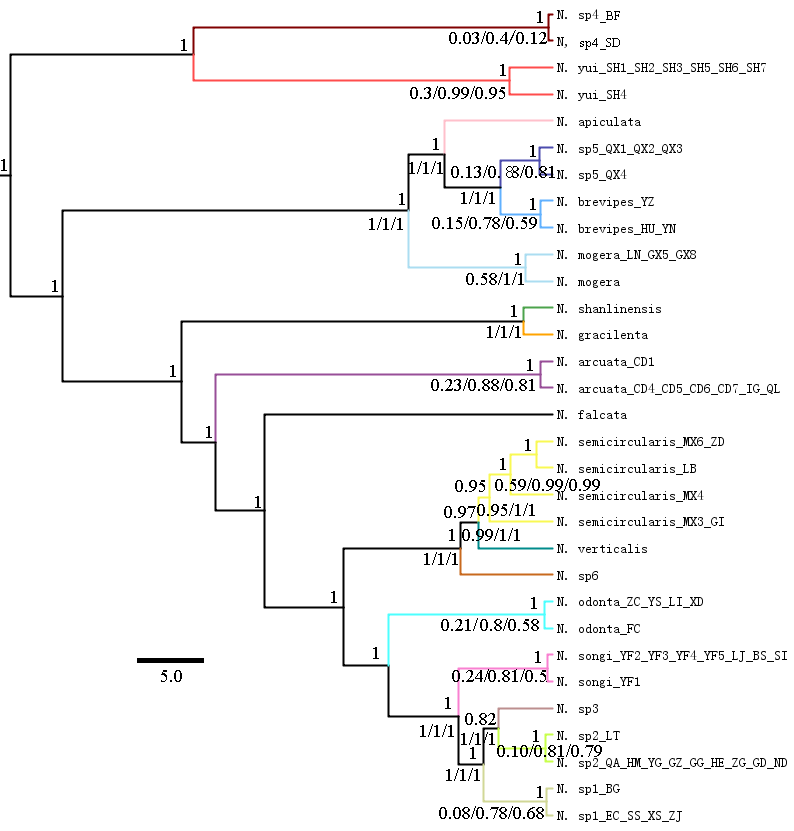

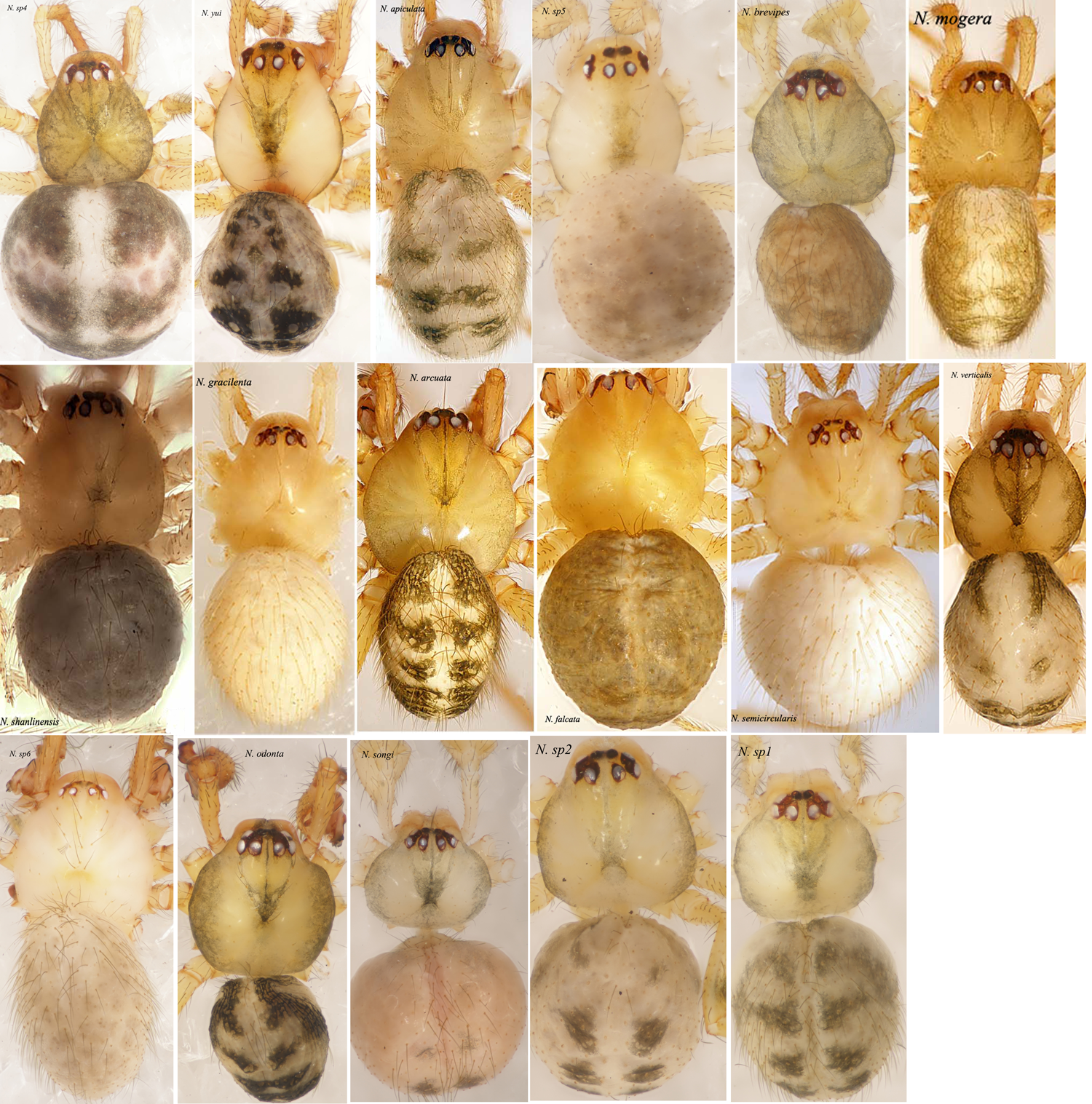


Additional file 4. **The figure above was the detailed results of the coalescent based species delimitation methods and below was photos of each species.** The species guide tree were constructed using *Beast. Tips of the tree, named by species names followed by an underscore and population codes, were operational taxonomic units testing in BPP. Posterior probabilities were shown below the nodes with prior combinations of θ~G(1, 10) and τ~G(1, 10), θ~G(2, 2000) and τ~G(2, 2000) and θ~G(2, 2000) and τ~G(1, 10). PP values lower than 0.95 across all prior combinations were omitted. Posterior probabilities of the guide species tree were shown above the nodes. Clade colors for each species were consistent with the sampling map. There is no photo for *N. sp3* because we only sample one specimen of it and this specimen was destroyed during DNA extraction.
