# Supplementary material for: Ancient lineage, young troglobites: recent colonization of caves by Nesticella spiders
Source: BMC Evol Biol. 2013 Sep 4;13:183. doi: 10.1186/1471-2148-13-183 (PMC3766682; doi:10.1186/1471-2148-13-183)
Supplement: Additional file 4 — The Bayesian gene tree reconstructed using mitochondrial (left) and nuclear (right) sequences. Outgroups Nesticus cellulanus and Theridion sp. were distantly related with the ingroup and not included. Numbers above nodes are posterior probabilities followed by maximum likelihood bootstraps and letters below nodes are the three main clade names. Colors of braches correspond to colors in Figure 1. [file 1471-2148-13-183-S4.doc]

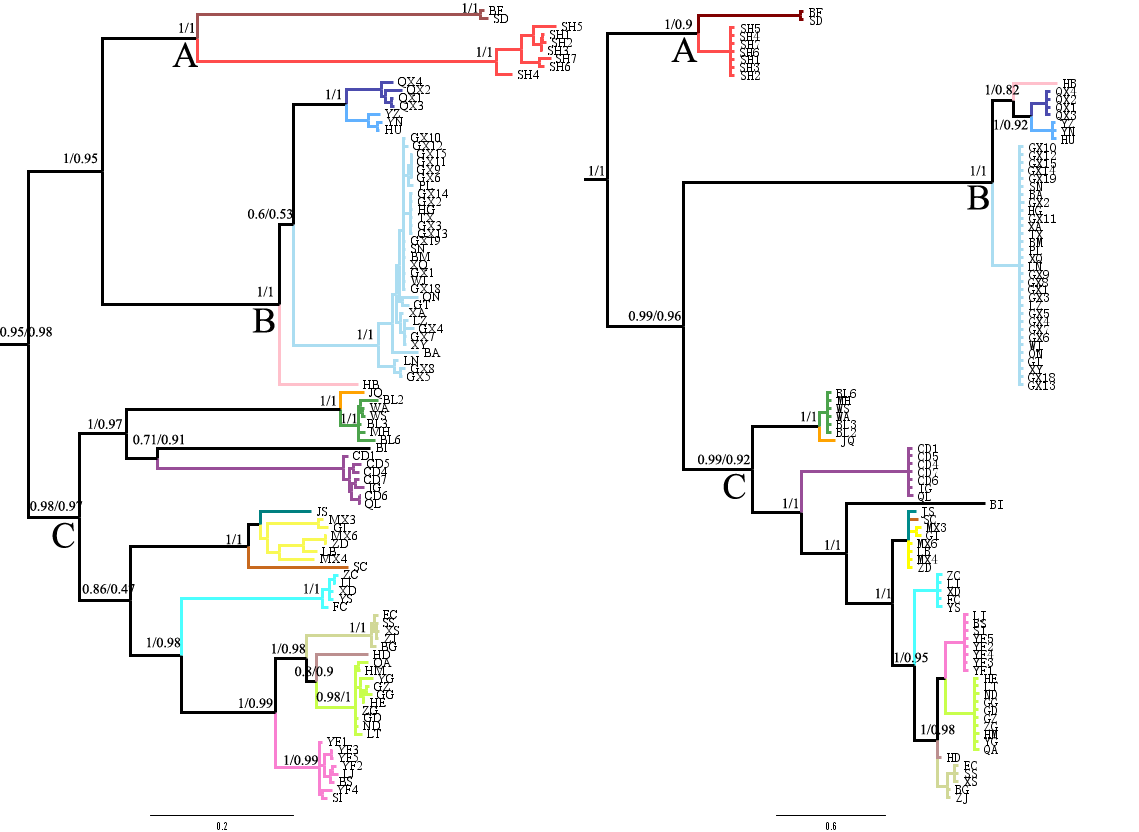


Additional file 3. The Bayesian gene tree reconstructed using mitochondrial (left) and nuclear (right) sequences. Outgroups *Nesticus cellulanus* and *Theridion* sp. were distantly related with the ingroup and not included. Numbers above nodes are posterior probabilities followed by maximum likelihood bootstraps and letters below nodes are the three main clade names. Colors of braches correspond to colors in Figure1.
