# Supplementary material for: Ancient lineage, young troglobites: recent colonization of caves by Nesticella spiders
Source: BMC Evol Biol. 2013 Sep 4;13:183. doi: 10.1186/1471-2148-13-183 (PMC3766682; doi:10.1186/1471-2148-13-183)
Supplement: Additional file 5: — Chronogram of Nesticella age divergences with 95% confidence intervals (blue bars). Numbers besides nodes were node ages. [file 1471-2148-13-183-S5.doc]

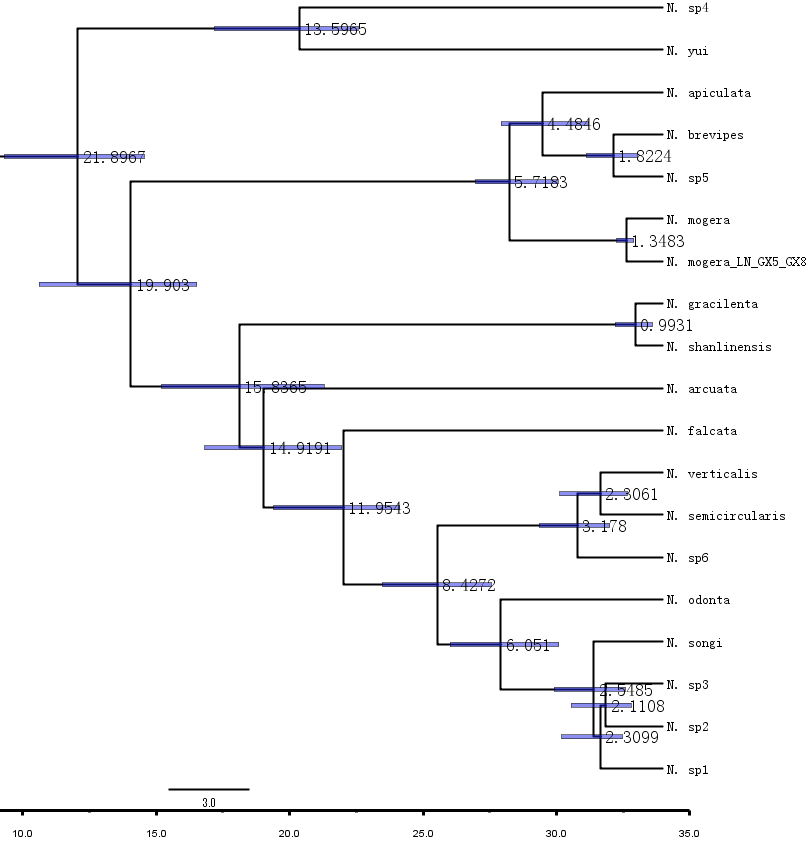


Additional file 5. **Chronogram of *Nesticella* age divergences with 95% confidence intervals (blue bars). Numbers besides nodes were node ages.**
